# Semen characteristics and second successful artificial insemination of Asian elephant (*Elephas maximus*) in Thailand

**DOI:** 10.14202/vetworld.2022.1246-1255

**Published:** 2022-05-22

**Authors:** Ampika Thongphakdee, Supalak Kiatsomboon, Sakhon Noimoon, Urarikha Kongprom, Itti Boonorana, Santhita Karoon, Jedsada Thawnern, Apichaya Sakulthai, Petthisak Sombutputorn, Manakorn Sukmak, Chainarong Punkong, Nikorn Thongtip

**Affiliations:** 1 Wildlife Reproductive Innovation Center, Conservation and Research Institute, Zoological Park Organization of Thailand under the Royal Patronage of H.M. the King, Chonburi 20110, Thailand; 2 Khao Kheow Open Zoo, Zoological Park Organization of Thailand under the Royal Patronage of H.M. the King, Chonburi 20110, Thailand; 3 Department of Farm Technology Management, Faculty of Agro-Industry, Panyapiwat Institute of Management, Nonthaburi 11120, Thailand; 4 National Elephant Institute, Forest Industry Organization, Lampang 52190, Thailand; 5 Department of Farm Resources and Production Medicine, Faculty of Veterinary Medicine, Kasetsart University, Kamphaeng Saen Campus, Nakhon Pathom 73140, Thailand; 6 Center for Agricultural Biotechnology, Kasetsart University, Kamphaeng Saen Campus, Nakhon Pathom 73140, Thailand; 7 Center of Excellence on Agricultural Biotechnology, Science and Technology Postgraduate Education and Research Department Commission on Higher Education, Ministry of Education (AG-BIO/PERDO-CHE), Bangkok 10903, Thailand; 8 Department of Large Animal and Wildlife Sciences, Faculty of Veterinary Medicine, Kasetsart University, Kamphaeng Saen Campus, Nakhon Pathom 73140, Thailand

**Keywords:** artificial insemination, Asian elephant, hormone, semen quality, sex accessory glands

## Abstract

**Background and Aim:**

As the number of wild Asian elephants (*Elephas maximus*) continues to decline, maintaining healthy populations under human care is vital. Male fertility assessment is essential for understanding the reproductive status, which can help to uncover underlying problems and improve the rate of pregnancy success. The objectives of this study in Asian elephants were as follows: (1) To investigate the semen characteristics; (2) to compare the relative seminal vesicle size and semen characteristics; (3) to compare the semen characteristics between good-motile (>60% progressive motility) and poor-motile (<60% progressive motility) ejaculates; and (4) to investigate the pregnancy success rate after artificial insemination (AI) with combined chilled and frozen semen.

**Materials and Methods:**

In total, 153 ejaculates were collected by manual rectal stimulation from 25 bulls. The volume, pH, sperm concentration, progressive motility, viability, morphology, and membrane integrity were investigated in each ejaculate. Assessment of accessory sex glands was conducted using transrectal ultrasonography to compare the relative seminal vesicle size and semen characteristics, and the bulls were divided into two groups according to the size of the ampulla (<7 or ≥7 cm^2^). For the comparison of good and poor-motile ejaculates and semen characteristics, the samples were divided into two groups: Good-motile (>60% progressive motility) and poor-motile (<60% progressive motility) ejaculates. Semen ejaculates for AI were collected from three bulls. The estrous cycles of four females were monitored using an enzyme immunoassay. Seven AI attempts were conducted using frozen and/or chilled semen by endoscopic visualization. AI was repeated 1 day before the luteinizing hormone surge, on the day of the surge, and 1 day after the surge. Pregnancy was confirmed by monitoring the serum progesterone profile and the abdomen and mammary glands changes.

**Results:**

From 153 ejaculates, the mean±standard error values of progressive motility, semen volume, sperm concentration, pH, and viability were 40.18%±2.28%, 40.94±3.86 mL, 1,205.58±62.26×10^6^ sperm/mL, 7.50±0.10, and 56.17%±1.96%, respectively. Comparing ampulla size and semen characteristics revealed that the bulls with ampullae of ≥7 cm^2^ yielded significantly larger volume ejaculates. However, there were no significant differences in sperm motility and concentration. The comparison of semen characteristics between good- and poor-motile ejaculates revealed that the former had significantly higher pH, viability, normal acrosomes, intact membranes, and normal head and tail morphology but often had a significantly lower volume and sperm concentration. From seven AI attempts in four females, one female had a confirmed pregnancy (14.3% pregnancy rate), and delivered a healthy live female baby weighing 128 kg at 21 months and 12 days of gestation. The baby is now 3 years old and in a healthy condition, with normally developing growth and behavior.

**Conclusion:**

The semen characteristics of Asian elephants can be used as the baseline reference for further applications. The ampullae size indicates semen quantity but not quality. Our success in producing an elephant calf from AI using frozen and chilled semen demonstrated that AI can be used as an alternative approach for the breeding management of Asian elephants. However, the semen of Asian elephants is of poor quality, especially in terms of membrane integrity; thus, the improvement in semen quality through intensive and careful management of elephant health and fertility remains a challenge for the future. Furthermore, a sperm bank should be established to develop sperm cryopreservation, which will be invaluable for improving the genetic diversity of the Asian elephant.

## Introduction

The Asian elephant (*Elephas maximus*) is endangered and has been listed in Appendix I of the Convention on International Trade in Endangered Species of Wild Fauna and Flora [1] since 1975. The species is distributed in 13 countries across South Asia and Southeast Asia, with an overall population of 48,323-51,680 in the wild and 15,000 in human care [2]. Thailand’s current population of legally designated captive elephants is estimated at 4,500 [3], which is more than its wild population of 3,168-3,440 [4]. The major threats to the survival of the species include habitat loss and fragmentation, human-elephant conflict, poaching, and the illegal trade of elephant body parts [2], all of which are driven by the rapid increase in the human population.

As the number of wild elephants continues to decline, maintaining a healthy *ex situ* population is vital. *Ex situ* conservation plays an important role in maintaining the diversity of captive wildlife populations of global importance [5]. Fertility assessment is essential for understanding the reproductive status. The criteria for estimating male fertility consider the normality of reproductive organs, including the accessory sex glands and semen characteristics. Semen characteristics of individuals/populations can help to determine underlying problems, allowing veterinary professionals to find solutions to improve the rates of a successful pregnancy. Moreover, the knowledge of sperm quality can be served as the reference data, which is useful for fertility investigation and other applications, that is, cryopreservation and artificial insemination (AI). These techniques help population managers to enrich captive or isolated wild elephant populations without removing valuable individuals from their natural habitat [6]. Although natural breeding is the first choice for sustaining the populations of endangered species, a high proportion of elephants fail to reproduce naturally [7], mainly due to difficulty controlling an aggressive male, behavioral incompatibility between some males and females [8], and sub/infertility [9]. In addition, transporting a bull elephant from one institution to another for natural breeding is expensive and increases the chance of injuries to the animal. Therefore, AI serves as an alternative tool for propagation in this species. Pregnancies have been reported following AI using either chilled [10] or frozen-thawed semen [11]; however, following the successful use of frozen semen in an AI pregnancy, the female aborted at 17 months of gestation [11]. Until now, there has been no report of the birth of elephant calves after AI using frozen-thawed semen.

The objectives of this study in Asian elephants were as follows: (1) To investigate the semen characteristics; (2) to compare the relative seminal vesicle size and semen characteristics; (3) to compare the semen characteristics between good-motile (>60% progressive motility) and poor-motile (<60% progressive motility) ejaculates; and (4) to investigate the pregnancy success rate after AI with combined chilled and frozen semen.

## Materials and Methods

### Ethical approval

Semen and blood sample collections and AI were carried out in accordance with Animal Care and Use Protocol and were approved under Project NRIIS 269284, issued by the Zoological Park Organization of Thailand under the Royal Patronage H.M. the King.

### Study period and location

The study was conducted from October 2014 to October 2019 at Wildlife Reproductive Innovation Center, Khao Kheow Open Zoo (KKOZ), Chonburi, the National Elephant Institute, Forest Industry Organization, Lampang, the Elephant Kingdom, Surin, and two private elephant camps in Chiang Mai.

### Chemicals

All chemicals in this study were purchased from Sigma Chemical Company (Sigma, St. Louis, MO, USA) unless stated otherwise.

### Animals

This study used 25 bulls (EM1-EM25) aged 10-49 years from KKOZ, Chonburi, the National Elephant Institute, Forest Industry Organization, Lampang, the Elephant Kingdom, Surin, two private elephant camps in Chiang Mai, and four females (FM1-FM4) aged 15-40 years from KKOZ. Three bulls (EM1-EM3) aged 25-41 years at KKOZ were the semen donors for AI. The elephants were fed mainly with grass, supplemented with pineapple tree, banana, and sugar cane during the day, and were allowed to roam freely or chained (25 m in length).

### Semen collection and evaluation

Semen collection was performed among the 25 bulls (153 ejaculates) using manual collection [12]. Before starting the semen collection, transrectal ultrasonography (LogicQ, GE Healthcare, United States) was used to perform assessments of the accessory sex glands (prostate, seminal vesicle, and ampulla).

Each ejaculate was immediately evaluated for volume, sperm concentration, progressive motility, sperm viability, and pH (using pH paper strips). Viability and head and acrosome morphology were assessed using eosin-nigrosin (6.7 g/L of eosin Y and 100 g/L of nigrosin in 9 g/L of sodium chloride), Diff-Quick, and Coomassie blue staining, respectively. For Coomassie blue staining, fixed sperm was centrifuged at 500× *g* for 5 min and then pellet was resuspended in 100 μL of 100 mM ammonium acetate. The suspended sperm was smeared on a glass slide and left to air dry. Once the slide was dry, it was placed in Coomassie blue stain solution (0.22% Coomassie blue G-250, 50% methanol, 10% acetic acid, and 40% water) for 2 min, washed with distilled water, and air-dried and examined under light microscopy at 40× magnification [13]. The sperm tail and concentration were assessed after fixation in 4% paraformaldehyde. The membrane integrity was assessed using the hypoosmotic swelling test (HOS test) [14], which is used to evaluate the functional integrity of the sperm plasma membrane. The assay is based on the transportation of fluid across an intact cell membrane under hypoosmotic conditions. The process continues until equilibrium is reached between the inside and outside of the cell. The influx of fluid serves to stretch the intact cell causing a bulge in the plasma membrane. The fibers (axonemal complex) of the sperm tail are covered by the plasma membrane. When the plasma membrane swells under hypoosmotic conditions, curling of the tail fibers occurs within the plasma membrane, which can be examined under a phase-contrast microscope (CX31 Upright Microscopy, Olympus, Japan). Spermatozoa with a chemically and physically intact membrane will show tail curling under hypoosmotic conditions, while spermatozoa with an inactive and non-intact membrane will not. Fresh semen osmolality was measured using an OSMOMAT^®^ 030 Automatic Cryoscopic Osmometer (Berlin, Germany).

For the comparison of the relative seminal vesicle size and semen characteristics, the bulls were divided into two groups according to the size of the ampulla: <7 cm^2^ (n=13) and ≥7 cm^2^ (n=12). The samples were divided into two groups for the comparison of good and poor-motile ejaculates and semen characteristics: Good-motile (>60% progressive motility, n=54) and poor-motile (<60% progressive motility, n=99) ejaculates.

For AI, semen samples from EM1 to EM3 were diluted with TEST yolk extender (5.54% Tes [N-tris(hydroxymethyl)methyl-2-aminoethane-sulphonic acid], 1.15% Tris-(hydroxymethyl)-aminomethane, 0.4% glucose, and 20% egg yolk) at a 1:1 ratio and cooled from room temperature (28-32°C) to 4°C at a rate of 1°C/min. The cooling system used a 28-32°C water jacket covering the semen tube, which was placed into a Styrofoam box that controlled the water temperature with ice and a thermometer at 4°C. It took 30 min for the samples to be cooled from 28-32°C to 4°C. After cooling, the semen was stored at 4°C in a temperature-controlled refrigerator (Biobase LCX-12L Portable Refrigerator, CE Group, China) and then warmed to 37°C before being used for AI.

### Semen freezing and thawing

Semen samples were frozen using the method outlined by Thongtip *et al*. [15]. Briefly, ejaculates were diluted (1:1) with TEST yolk extender supplemented with 20% egg yolk at 28-32°C. The semen mixture was cooled from 28-32°C to 4°C at a rate of 1°C/min using the technique described above. After cooling, the semen was stored at 4°C in a temperature-controlled refrigerator. Then, an equal volume of TEST yolk extender containing 10% (v/v) glycerol was divided into four parts and each part was added to the semen mixture every 15 min and further equilibrated at 4°C for 1 h. The equilibrated mixture was loaded into 0.5-mL labeled plastic straws (Kruuse, Ltd., Leeds, United Kingdom). Before loading, the straws were placed in the temperature-controlled refrigerator to maintain them at a temperature equal to that of the semen. The straws were sealed with sealing powder (Arstm, Chino, California, United States), and kept at 4°C for around 10 min before placing on a stainless-steel rack in a Styrofoam cooler containing liquid nitrogen. The straws were held 2.5-cm above the liquid nitrogen for 10 min and then plunged into liquid nitrogen for storage until required for AI. For thawing, the straws were placed in a 37°C water bath for 30 s. The semen samples were expelled into a 50-mL tube and kept in a 37°C water bath. An aliquot (10 μL) of the thawed semen was removed and placed on a pre-warmed microscopic slide to assess sperm total motility and progressive motility using light microscopy (CX31 Upright Microscopy, Olympus).

### AI and pregnancy monitoring

The estrous cycle was monitored using established hormone assays. Whole blood (10 mL) was collected from an ear vein weekly. When the progesterone concentrations decreased to baseline (during the follicular phase), blood was collected daily (at the same time each day) until the concentrations increased again. Blood samples were allowed to clot at 28-32°C for 1-2 h and then centrifuged at 1500×*g* for 5 min to separate the serum from blood cells. Serum samples were transferred into 1.5-mL microtubes and stored at −20°C until analysis.

Serum progesterone was analyzed using an enzyme immunoassay (EIA) as described by Thitaram *et al*. [16]. The EIA used a monoclonal antibody against progesterone (1:53,000; Quidel clone #425 Quidel clone#425; supplied by C. Munro, University of California, Davis, California, United States), which has been shown to cross-react with a variety of reduced pregnancy in the serum and excreta of a wide range of species, including elephants [17]. Bound progesterone was visualized using a horseradish peroxidase-conjugated antibody to progesterone (1:75,000; C. Munro, University of California, Davis, United States) and quantitation was achieved by comparing the level of color development to that of known progesterone standards using an enzyme-linked immunosorbent assay plate reader (Tecan^®^ sunrise absorbance reader; Tecan Austria GmbH, Austria). The sensitivity of the assay was 0.016 ng/mL, the average inter-assay coefficient of variation (CV) for the high and low concentration controls was 7.32 and the average intra-assay CV for the high and low concentration controls was 5.15.

Serum luteinizing hormone (LH) was analyzed using the EIA as described by Thitaram *et al*. [16]. The LH EIA used a monoclonal anti-bovine LH antiserum (518-B7), a biotin-conjugated ovine LH label, and bovine LH (NIH-LH-B10; AFP-5551B) standards. This monoclonal antibody has been validated to detect LH in Asian elephants [10]. The LH label was prepared using an EZLinkTM Sulfo-NHS-LC-Biotinylation kit (Pierce, Rockford, Illinois, United States). The sensitivity of the assay was 0.039 ng/mL, the average inter-assay CV for the high and low concentration controls was 9.98, and the average intra-assay CV for the high and low concentration controls was 8.25.

To facilitate timed AI, we detected the first LH surge, previously identified as the anovulatory LH surge, which predictably occurs 3 weeks before the ovulatory LH (ovLH) surge. On the day of insemination, the female was subjected to a standing immobilization using 0.04 mg/kg xylazine HCL (Xylaz, Farvet Laboratories BV, Bladel, the Netherlands) administered intravenously. Seven AI attempts were conducted from 2015-to 2018. Endoscopic visualization used a fiber-optic video endoscope, video processing circuit, and light source of PENTAX (EPM3300 and EC3830 fz) to guide the insemination catheter into the external os of the cervix and the semen was deposited. This was repeated 1 day before the LH surge, on the day of the surge, and 1 day after the surge. Briefly, an external electric air pump or oxygen tank was connected to the working channel of the endoscope to facilitate inflation (enlarging) of the vestibular, vaginal, and external os of the cervical canal. With the tip of the endoscope at the external os of the cervix, the inflated air tube was removed and the insemination catheter was then passed through the working channel of the endoscope. For the seven AI attempts, 20-150 mL of chilled semen was deposited per insemination in all females (FM1-FM4). In addition, females FM 3 and FM 4 were inseminated with 20 and 30 mL of frozen-thawed semen, respectively, on the 1^st^ day of insemination. The ranges of the percentage of progressive motility and viability after dilution before AI were 20-80% and 22-97%, respectively. The range of sperm concentration after dilution before AI was 22.5-1,363×10^6^ sperm/mL. During the injection process, signs of semen reflux were monitored until all the semen had been inseminated. Then, the catheter and endoscope were gently removed. Pregnancy was confirmed by monitoring the serum progesterone profile and the changes in the abdomen and mammary glands. Serum progesterone was analyzed using an EIA as described previously [15].

### Parentage testing

Genomic Deoxyribonucleic acid (DNA) was isolated from whole blood samples collected from the male, female, and AI calf using a high pure polymerase chain reaction (PCR) Template Preparation Kit (Roche Diagnostics, Basel, Switzerland). Twelve primer pairs of microsatellite markers were used for PCR: EMU04, EMU07, EMU14, EMU15, EMU17, LA2, LafMS03, LafMS05, LafMS06, LafMS08, FH60, and FH 94 [18]. Extracted DNA was amplified in 25 μL reaction volumes containing 2.5 μL Supertaq 10× buffer (Applied Biosystems, Foster City, California, United States), 5 mM dNTP (1 μL) (Qiagen Inc., Valencia, California, United States), 5 pmol primers (1 μL), 0.4 U of Super Taq DNA polymerase (0.25 μL) (Applied Biosystems), 50 ng of extracted DNA (1 μL), and Milli Q water (18.25 μL). PCR was performed in a Peltier Thermal Cycler 220 (M J Research Inc., Waltham, Massachusetts, United States). PCR consisted of a single denaturation step at 94°C for 2 min, followed by 34 cycles of 94°C denaturation for 30 s, 30 s of primer annealing with the specific temperature of each primer, and then 30 s of primer extension at 72°C, followed by a single extension of 72°C for 2 min. Positive PCR products generated by specific primers of microsatellite markers were fractionated on a 1.5% agarose gel (Seakem^®^ LE agarose, BioWhittaker Molecular Applications, Rockland, Maine, United States). The PCR products were submitted for fragment analysis using ABI3730XL at 1^st^ Base (Malaysia).

### Statistical analysis

Statistical analyses were performed using Statistical Package for the Social Sciences v 20.0 software (IBM Corporation, Armonk, NY, United States). The independent t-test was used to analyze the mean differences of the ampulla size (<7 or ≥7 cm^2^) on seminal vesicle size and semen characteristics (volume, motility, and concentration) together with the mean differences of the quality of motile sperm (good-motile-motility>60% and poor-motile-motility<60%) on the semen characteristics, including the semen volume, semen concentration, pH, viability, intact acrosome, membrane function integrity, normal head and tail morphology, and osmolality. The results are presented as the mean±standard error (SE) at a p<0.05, which was considered to indicate a statistically significant difference. Before determining the statistical difference, the population variance (equal or unequal variance) between the number of samples in each treatment group and each semen characteristic parameter was tested by Welch’s t-test.

## Results

### Semen characteristics

During the 3-year study period, 153 ejaculates were collected from 25 bulls through manual collection. All bulls produced predominantly poor-quality ejaculates, especially regarding progressive motility, membrane function integrity, and tail morphology (Table-1). The mean±SE values of the progressive motility, semen volume, sperm concentration, pH, and viability were 40.18%±2.28%, 40.94±3.86 mL, 1,205.58±62.26 × 10^6^ sperm/mL, 7.50±0.10, and 56.17%±1.96%, respectively. The mean±SE values of the percentages of the intact acrosome, membrane function integrity, normal head, and normal tail were 67.96%±1.66%, 35.78%±2.08%, 69.29%±1.60%, and 49.10%±1.64%, respectively. The mean±SE of osmolality was 302.00±25.21 mOsm. Post-thawed sperm had low motility (0-40%). Only post-thawed samples with 15-40% motility were used for AI.

**Table 1:** Fresh semen quality of 153 ejaculates from 25 bulls.

Parameter	All ejaculates

	Mean±SE (range)
Progressive motility (%)	40.18±2.28 (0-90)
Volume (mL)	40.94±3.86 (0.35-243)
Concentration (×10^6^/mL)	1,205.58±62.26 (0-3,800)
pH	7.50±0.10 (4-10)
Viability (%)	56.17±1.96 (0-98)
Normal acrosome (%)	67.96±1.66 (23-93)
Membrane intact (%)	35.78±2.08 (0-89.5)
Normal head (%)	69.29±1.60 (7-98)
Normal tail (%)	49.10±1.64 (1-90)
Osmolality (mOsm)	302.00±25.21 (115-595)

SE=Standard error

### Comparison of the relative seminal vesicle size and semen characteristics

Due to the elephant positive reinforcement training program, transrectal ultrasonography of the prostate glands, ampulla glands, and seminal vesicles was successfully performed before starting semen collection in all bulls. One of the criteria for estimating the success of semen collection was the size of hypo-echoic images inside the ampulla and seminal vesicles. An enlargement of the hypo-echoic area inside the lumen of both glands suggested the presence of fluid within. The prostate glands appeared like a cluster of grapes with lumens filled with hypo-echoic fluid. However, it was difficult to visualize the glands when the prostate gland was not adequately stimulated (inactive state). According to the size of the ampulla (<7 or ≥7 cm^2^) and comparing the relative seminal vesicle size and semen characteristics, bulls with ampullae of ≥7 cm^2^ yielded significantly larger volume ejaculates. However, there were no significant differences in sperm motility and concentration (Table-2).

**Table 2:** Comparison of size of seminal vesicle and semen characteristics between small and big ampulla.

Parameter (Mean±SE)	Group 1 (n=13) (<7 cm^2^)	Group 2 (n=12) (≥7 cm^2^)
Ampulla (cm^2^)	3.75±0.47^b^	15.73±3.15^a^
Seminal vesicle (cm^2^)	7.38±1.19^a^	11.37±2.48^a^
Volume (mL)	22.77±2.77^b^	59.56±9.02^a^
Motility (%)	34.83±7.37^a^	22.98±6.98^a^
Concentration (10^6^/mL)	1,670.40±233.25^a^	1,787.00±142.57^a^

Values (mean±SE) with different lowercase superscripts in the same row are significantly (p<0.05) different. SE=Standard error

### Comparison of semen characteristics between good- and poor-motile ejaculates

The comparison of semen characteristics between good-motile (>60% progressive motility) and poor-motile (<60% progressive motility) ejaculates revealed that the former often had a lower volume and sperm concentration (Table-3). Good-motile ejaculates also had a higher pH, viability, normal acrosome, membrane function integrity, and normal head and tail morphology.

**Table 3:** Comparison of semen characteristics between good-motile (≥60% progressive motility) and poor-motile (<60% progressive motility) ejaculates.

Semen parameter	Good-motile (54 ejaculates; 9 bulls) Mean±SE (range)	Poor-motile (99 ejaculates; 22 bulls) Mean±SE (range)
Motility (%)	71.02±1.13^a^ (60-90)	23.36±1.98^b^ (0-55)
Volume (mL)	26.58±3.59^b^ (0.5-119)	48.77±5.49^a^ (0.35-243)
Concentration (×10^6^/mL)	1,014.81±76.72^b^ (155-2,820)	1,309.62±85.10^a^ (0-3,800)
pH	8.02±0.10^a^ (7-10)	7.22±0.13^b^ (4-10)
Viability (%)	76.07±1.81^a^ (44.5-97.5)	45.32±2.18^b^ (0-87)
Normal acrosome (%)	74.00±2.26^a^ (45-93)	64.15±2.17^b^ (23-93)
Membrane intact (%)	53.47±2.86^a^ (20-89.5)	26.85±2.19^b^ (0-87)
Normal head (%)	77.52±2.10^a^ (23-98)	64.81±2.06^b^ (7-98)
Normal tail (%)	57.79±2.51^a^ (11-90)	44.37±1.98^b^ (1-87)
Osmolality (mOsm)	255.67±23.05^a^ (115-335)	319.38±29.58^a^ (116-595)

Values (mean±SE) with different lowercase superscripts in the same row are significantly (p<0.05) different. SE=Standard error

### AI and pregnancy success

**Figure-1:**
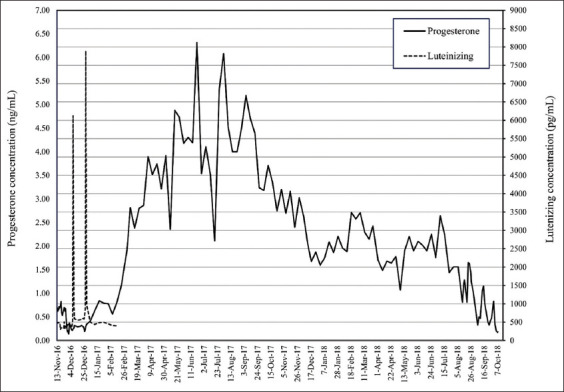
Profiles of serum LH and progesterone determined by EIA from anLH surge throughout gestation. EIA=Enzyme immunoassay, anLH=Anovulatory luteinizing hormone.

**Figure-2:**
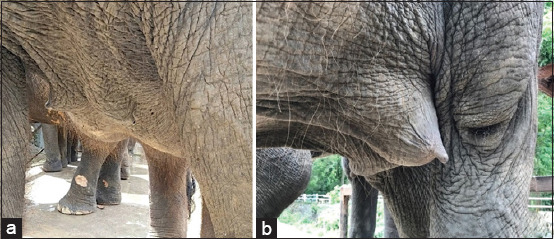
Characteristic of mammary glands before AI (a) and at 18 months of gestation (b). AI=Artificial insemination.

**Figure-3:**
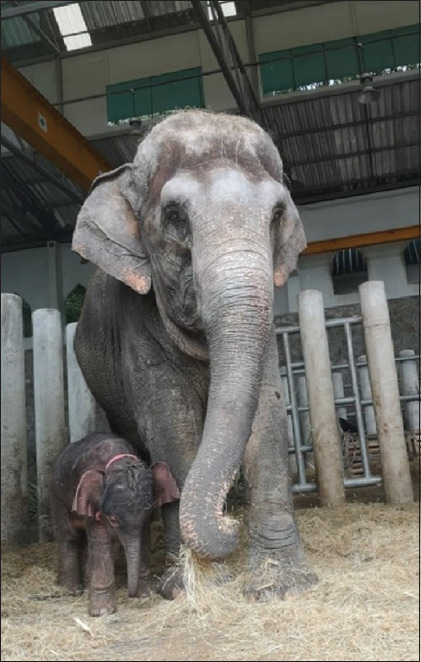
One-day-old female AI calf and her mother (FM4). AI=Artificial insemination.

From a total of seven AI attempts in four females, one female (FM 4) had a confirmed pregnancy (14.3% pregnancy rate). The pregnant female was inseminated for 3 days consecutively, with frozen semen on the 1^st^ day and chilled semen on the next 2 days. The progressive motility of frozen-thawed semen was 20% (using 30 mL of 100×10^6^ sperm/mL for AI) and that of chilled semen was 50-60% (using 100-150 mL of 260-280×10^6^ spermatozoa for AI), as shown in Table-4. The highest ovLH surge was 7.61 ng/mL, which was recorded in two females (FM2 and FM4) during the second and sixth attempts, although only FM4 became pregnant (Table-4). The progesterone profile of FM4 increased after AI and was maintained throughout gestation in the pregnant female (Figure-1) but gradually declined to the baseline in the non-pregnant females (FM1, FM2, and FM3). In the pregnant elephant, the serum progesterone levels started to decline to the baseline a couple of days before parturition. Enlargement of mammary glands was observed during the last trimester of gestation (Figure-2). The FM4 female delivered a healthy live female baby weighing 128 kg at 21 months and 12 days of gestation (Figure-3). The calf, named “SanRak” (meaning lots of love), appeared healthy and exhibited normal suckling behavior.

**Table 4:** AI pairings, concentration of OvLH, semen type, and semen qualities of AI trials.

S. No.	AI		OvLH (ng/mL)	Day 1					Day 2					Day 3					Preg. result
			
	♀	♂		Semen type	Vol. (mL)	Moti. (%)	Via. (%)	Conc. (10^6^ sp/mL)	Semen type	Vol. (mL)	Moti. (%)	Via. (%)	Conc. (10^6^ sp/mL)	Semen type	Vol. (mL)	Moti. (%)	Via. (%)	Conc.(10^6^ sp/mL)	
1.	FM1	EM1	3.42	Chilled	20	30	52	200	Chilled	20	80	90	60	Chilled	20	70	90	77	No
2.	FM2	EM2	7.61	Chilled	20	50	69	300	Chilled	20	80	62	103	-	-	-	-	-	No
3.	FM1	EM2	0.36	Chilled	20	80	97	54	Chilled	20	40	64	150	Chilled	20	70	52.5	165	No
4.	FM2	EM3	6.40	Chilled	50	50	22	810	Chilled	46	70	59	400	Chilled	20	70	73	22.5	No
5.	FM3	EM2	0.36	Frozen	20	20	50	200	Chilled	20	60	60	900	-	-	-	-	-	No
6.	FM4	EM3	7.61	Frozen	30	20	56	100	Chilled	150	50	67	520	Chilled	100	60	49	295	Yes
7.	FM2	EM2	2.19	Chilled	20	50	80	1,363	Chilled	40	60	87	760	Chilled	20	50	79	300	No

OvLH=Ovulatory luteinizing hormone, AI=Artificial insemination, Vol.=Volume, Via.=Viability, Moti.=Motility, Conc.=Concentration, spz/mL=sperm/mL, Preg.=Pregnancy

For parentage testing, all 12 microsatellite markers were successfully amplified in the candidate father, mother, and AI calf. The microsatellite profile of the AI calf revealed five and seven homozygous and heterozygous alleles, respectively. All of the alleles of the AI calf were shared by the EM3 father, with no missing genotypes of the mother or father. Thus, the results confirmed the Mendelian inheritance of all markers, demonstrating that the AI calf was sired by the EM3 male. The microsatellite profiles of the AI calf and the parents (EM3 and FM4) are shown in Table-5.

**Table 5:** Allele sizes of 12 microsatellite markers of AI baby family.

Microsatellite markers	Allele sizes					

	Father (EM3)		Mother (FM4)		AI calf	
EMU04	104	104	104	104	104	104
EMU07	101	109	101	101	101	109
EMU14	131	139	131	137	137	139
EMU15	153	153	143	153	153	153
EMU17	121	129	129	135	129	129
LafMS03	149	149	137	149	137	149
LafMS05	145	153	153	157	145	157
LafMS06	146	146	146	150	146	146
LafMS08	188	188	178	190	188	190
FH60	152	152	154	158	152	158
FH94	221	223	217	221	217	223
LA2	223	229	229	229	229	229

AI=Artificial insemination

## Discussion

The study demonstrated the semen characteristics of a large number of Asian elephants (153 ejaculates from 25 bulls). The poor fresh semen quality obtained from the manual collection in the present study supports the findings of our previous study [9] and those of others [19-21]. However, until now, there has not been a more suitable method to collect semen from an elephant. Kiso *et al*. [19] reported that fewer than 13% of all attempts to collect semen in Asian elephants had resulted in ejaculates containing >60% motile spermatozoa. The present study revealed that only 35% (54 from 153 ejaculates) of ejaculates contained >60% motile spermatozoa. Nevertheless, with more samples of poor quality, some samples had good-motile sperm, which we used for the cryopreservation study. Compared to our previous study [15], the progressive motility of frozen-thawed quality was poorer than that observed previously, although the reasons for this remain unclear. One reason may be the poor membrane function reflected by the membrane function integrity using the HOS test. Our previous study used the viability or membrane intact through eosin-nigrosin staining in the semen evaluation protocol, showing acceptable results for frozen-thawed viability but unacceptable results from the HOS test. It has been reported that there is a relationship between poor post-thaw sperm motility and a poor HOS test percentage [22]. The conventional sperm characteristics assessment, including the concentration, motility, normal morphology, and viability, of pre-frozen semen samples were of limited value in predicting the cryosurvival of human spermatozoa [22]. In the present study, the poor membrane function integrity may explain the poor post-thawed progressive motility. Consequently, in future elephant sperm assessments, the HOS test should be conducted in parallel with eosin-nigrosin staining. Besides, the investigation into the occurrence of sperm DNA fragmentation in Asian elephants showed that the ejaculates were relatively poor quality, suggesting that Asian elephant spermatozoa are more susceptible to DNA fragmentation than the spermatozoa of other mammals [23]. Other factors, such as the chemistry and protein (lactotransferrin) profiles in seminal plasma, seem to play a role in sperm function and physiology, including sperm motility [24]. This indicates that more research is needed to overcome the poor semen quality since there are many factors involving semen quality, that is, health, reproductive, and mental conditions. For cryopreservation, the media used in the study consisted of simple components including glucose, egg yolk, TES buffer, antibiotic, and glycerol [15] without any supplements such as antioxidants and amino acids. Arnold *et al*. [25] used Berliner Cryomedium extender with Orvus Equex Paste and alpha-tocopherol for freezing elephant sperm, which resulted in better frozen-thawed quality than that observed in the present study. Interestingly, the protamine sequence of elephant sperm contains a few cysteine residues, which are fragile for cryopreservation by the rapid DNA fragmentation process [26]. Supplementation of cysteine in the semen extender may help to support and stabilize the chromatin structure in elephant spermatozoa by the establishment of disulfide crosslinks between protein molecules. Besides, supplementation of sericin, an antioxidant agent, improved semen quality after cryopreservation, as shown in a previous study in buffalos [27]. Thus, we plan to add other antioxidants, such as sericin and cysteine, in the semen extender in the future. In the present study, we divided the bulls into two groups according to the size of the ampulla (<7 or ≥7 cm^2^) and compared the relative seminal vesicle size and semen characteristics. Similar sperm motility and concentration were found in both groups. However, bulls with ≥7 cm^2^ ampullae yielded significantly larger volume ejaculates. These findings indicate that ultrasonography of sex accessory glands before semen collection not only helps to diagnose the possible abnormality of the organs but also predicts the quantity of the semen. However, the size of the ampulla does not represent the semen quality.

**Figure-4:**
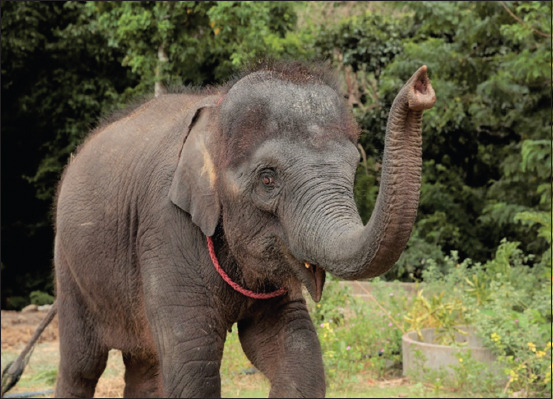
SanRak at 2 years and 10 months old.

Other studies showed successful pregnancy in Asian and African elephants from AI using good-to-excellent quality frozen-thawed semen [6,11]. The present study showed that low-quality semen could also be used to achieve a successful AI pregnancy. We suggest that low-quality semen is usable for AI when good quality semen is not available. However, successful pregnancy depends on the female being a proven fertile breeder. In the present study, the female that conceived (“Jim” or FM4) was a proven breeder (achieved three pregnancies and gave birth to two live calves by natural mating). The study was her first round of AI and it was a successful pregnancy followed by the delivery of a healthy (normal in health and behavior and had a normal suckling reflex) live calf weighing 128 kg, born on October 8, 2018, at 21 months and 12 days of gestation (Figure-3). This is the second live calf born using AI (using frozen and chilled semen) in Thailand; now, she is 3-years-old and in a healthy condition, developing normal growth and behaviors (Figure-4). The first calf born using AI (chilled semen) in Thailand is now 15 years old and remains healthy [11]. Other attempts using frozen semen for AI have shown pregnancy success in African [6] and Asian [11] elephants, but the delivery of a live calf has never been reported.

## Conclusion

The semen characteristics of Asian elephants reported in this study can be used as the baseline reference for further applications. The ampullae size indicates semen quantity but not quality. Our success in producing an elephant calf from AI using frozen and chilled semen demonstrates that AI can be used as an alternative approach for the breeding management of Asian elephants. However, Asian elephant semen is of poor quality, especially in terms of membrane integrity. A future challenge is to improve semen quality through the intensive and careful management of elephant health and fertility. Furthermore, a sperm bank should be established to develop sperm cryopreservation, which will be invaluable for improving the genetic diversity of the Asian elephant.

## Authors’ Contributions

AT: Conceptualization, formal analysis, project administration, and drafted and revised the manuscript. AT and NT: Methodology, data curation, visualization. SK, SN, UK, IB, SaK, JT, AS, PS, MS, and CP: Investigation. AT and SK: Software and the first draft of the manuscript. NT: Supervision. AT and NT: Validation. All authors have read and approved the final manuscript.
